# Deep Learning for Joint Pilot Design and Channel Estimation in MIMO-OFDM Systems

**DOI:** 10.3390/s22114188

**Published:** 2022-05-31

**Authors:** Xiao-Fei Kang, Zi-Hui Liu, Meng Yao

**Affiliations:** Affiliation College of Communication and Information Engineering, Xi’an University of Science and Technology, Xi’an 710054, China; zh_liu0619@163.com (Z.-H.L.); shirley980912@126.com (M.Y.)

**Keywords:** autoencoder, channel estimation, conditional generative adversarial network, MIMO-OFDM, pilot design

## Abstract

In MIMO-OFDM systems, pilot design and estimation algorithm jointly determine the reliability and effectiveness of pilot-based channel estimation methods. In order to improve the channel estimation accuracy with less pilot overhead, a deep learning scheme for joint pilot design and channel estimation is proposed. This new hybrid network structure is named CAGAN, which is composed of a concrete autoencoder (concrete AE) and a conditional generative adversarial network (cGAN). We first use concrete AE to find and select the most informative position in the time-frequency grid to achieve pilot optimization design and then input the optimized pilots to cGAN to complete channel estimation. Simulation experiments show that the CAGAN scheme outperforms the traditional LS and MMSE estimation methods with fewer pilots, and has good robustness to environmental noise.

## 1. Introduction

MIMO-OFDM technology can effectively utilize resources in three dimensions of time, frequency, and space to greatly improve the spectral efficiency, power efficiency, and transmission rate of the system. It has become the core technology of broadband wireless communication systems. Acquiring accurate channel state information (CSI) through channel estimation is a prerequisite for realizing the huge potential of MIMO-OFDM technology, and it is also an important basis for realizing precoding, resource allocation, signal detection, indoor positioning, physical layer security, and so on [1].

Depending on whether pilot signals are needed, channel estimation can be classified as blind channel estimation and pilot-based channel estimation. Blind channel estimation does not require pilot signals, which perform channel estimation through the second-order or high-order statistical information of the received signal. In [2], a blind channel estimation algorithm is proposed that used the statistical information on the average power of the received signal to convert the average power of the received signal into a quadratic equation including the channel gain. The user terminal can estimate the massive MIMO downlink channel gain without any downlink pilot resources, but the algorithm is only suitable for time division duplex (TDD) systems and it does not take advantage of the channel sparse characteristics and the estimation accuracy performed poorly. A blind estimation algorithm based on expectation maximization (EM) is proposed for massive MIMO systems in [3], which utilized the sparse characteristics of the channel in the angular domain to improve the channel estimation accuracy but requires a large amount of computation. Blind channel estimation has the advantages of less prior information and high spectral efficiency, but its application is seriously limited to low channel estimation accuracy, high complexity, and poor real-time performance. The pilot-based channel estimation method is to insert pilot symbols into the transmitted signals, and the receiver implements channel estimation according to the received pilot signals. Despite the loss of spectral efficiency compared with blind channel estimation, it is widely used due to its simplicity of implementation. Commonly used pilot-based channel estimation algorithms include least square (LS) [4] and minimal mean square error (MMSE) [5]. The LS algorithm is a simple interpolation-based method and suffers from poor performance due to ignoring the handling of noise. The MMSE algorithm considers the complete channel statistics and noise variance, so the estimation accuracy is better than that of the LS algorithm, but it needs to invert the channel correlation matrix, which has high computational complexity and requires prior channel statistics. In [6], an approximated linear version of the MMSE (ALMMSE) is proposed in fast fading channels whose complexity is much less than the original MMSE due to reducing the size of the correlation and the filtering matrix.

MIMO-OFDM system usually includes multiple sub-carrier channels and many transceiver antenna pairs, so numerous channel parameters need to be estimated, especially for massive MIMO system, traditional pilot-based channel estimation algorithms will lead to huge pilot and feedback overhead, and the severe loss of performance in both spectral efficiency and complexity limit the application. Considering the sparse characteristics of massive MIMO-OFDM channels, some time-domain sparse channel estimation algorithms have been proposed, among which the compressed sensing (CS) algorithm [7,8] can simultaneously estimate the significant tap positions and their corresponding channel coefficient, and it is favored to use fewer pilots to obtain better estimation performance. In MIMO-OFDM channel estimation based on compressed sensing, the performance of the reconstruction algorithm depends on the recovery matrix, which is determined by the pilot positions. Therefore, how to effectively exploit the channel block sparse feature to jointly optimize pilot locations and symbols to ensure reconstruction accuracy is a challenge for such algorithms.

With the in-depth study of massive MIMO-OFDM channels, the performance improvement of existing optimization algorithms based on ideal assumptions and model approximation tend to saturate in increasingly complex application scenarios. Channel estimation based on deep learning provides a new idea to break through this bottleneck. Deep learning adopts big data offline training and real-time data online rapid update mode. On the one hand, its powerful feature extraction and nonlinear mapping capabilities can effectively improve the performance of channel estimation in complex scenarios. On the other hand, it chooses to balance the high complexity of online testing with offline training complexity to achieve higher operating efficiency. In [9], a DL-based channel estimation and direction-of-arrival (DOA) estimation solution was proposed for massive MIMO systems, where the DNN was exploited to learn the statistical characteristics of wireless channels and the spatial structure in the angle domain. In [10], an LDAMP deep learning network was put forward based on the denoising-based approximate message passing (D-AMP) algorithm for massive MIMO millimeter wave channel estimation. The channel matrix was equivalent to two-dimensional images used as network input, and residual learning was used to reduce training time and improve channel estimation accuracy. In [11], a channel estimation algorithm was proposed based on MMSE combined with deep learning, seeking to improve estimation accuracy and reduce complexity. In [12], a CsiNet was created based on a convolutional neural network (CNN) to realize the compression feedback and reconstruction of CSI. The authors of [13], dished an improved scheme CsiNet-LSTM, which take advantage of the time-varying MIMO channel time correlation, and the long short time memory networks (LSTM) were used for channel state information feedback and reconstruction. The authors of [14] applied meta-learning to OFDM system channel estimation for the first time and proposed RoemNet network architecture. The meta-learning machine only used a small number of pilots to complete new channel learning tasks and could reduce the impact of Doppler spread.

In summary of the above, Considering the influence of pilot design and estimation algorithm on the channel estimation result in pilot-based channel estimation, and taking into account that in complex application scenarios, the powerful feature extraction and nonlinear mapping capabilities of deep learning can effectively improve the performance of channel estimation. In this paper, we propose a hybrid network architecture called CAGAN that combines Concrete AE and cGAN organically. Using the proposed CAGAN deep learning network enables accurate channel estimation based on pilot design. The main contributions of this work are listed as follows:

- Compared with the deep learning-based channel estimation methods in the existing references [9,10,11,12,13,14], this paper applies a hybrid deep learning framework to integrate the two functions of pilot design and channel estimation. Pilot optimization and channel estimation are performed simultaneously during offline training.
- In order to obtain the optimal pilot design and higher channel estimation accuracy, this paper adopts the Concrete selector layers and the fully connected neural networks as the encoder and decoder respectively, the decoder also acts as the generator of the conditional generative adversarial network, and we use the combined loss function to constrain the optimization direction of the entire network.
- Finally, the simulation results show that the method proposed in this paper can significantly improve the channel estimation accuracy and save the pilot overhead in the MIMO-OFDM systems.

The rest of this paper is organized as follows: Section 2 introduces the principle of the proposed network structure, Section 3 explains the mathematical system model, simulation results are presented in Section 4 and finally, Section 5 concludes the paper.

## 2. Network Architecture and Implementation Principles

**Figure 1:**
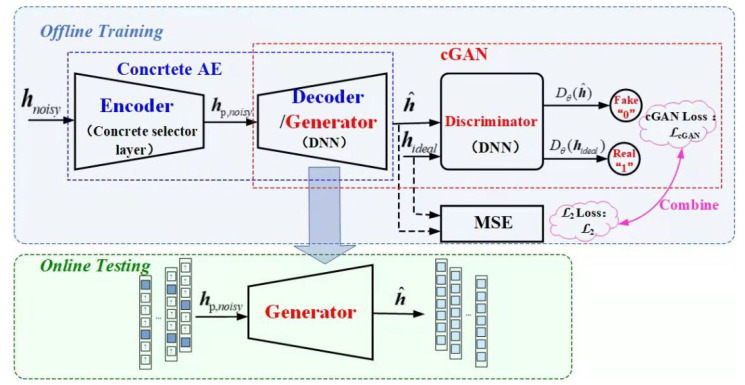
Architecture of the proposed CAGAN approach.

The proposed CAGAN network is an organic mixture of an autoencoder based on Concrete distribution (concrete AE) and a conditional generative adversarial network (cGAN). The decoder of Concrete AE and the generator of cGAN are combined into one, using the same deep neural network (DNN) implementation, as shown in Figure 1.

As shown in the figure, offline training consists of two parts: Firstly, concrete AE is used for pilot optimization design, in which the concrete selector layer selects the optimal sub-feature set to obtain the optimized pilots channel; secondly, the cGAN network implements channel estimation, specifically, the optimized pilots are input to the generator to generate the estimated channel, the estimated and the real channel are simultaneously input into the discriminator. The discriminator and the generator achieve a Nash equilibrium through multiple cycles of alternate training. At this time, the cGAN network converges, and the estimated value and the real value tend to be consistent in distribution. In addition, in order to ensure the correct direction of the generator optimization, the cGAN loss function additionally adds a mean square error (MSE) loss function between the estimated channel and the real channel. After the training of the CAGAN network is completed, during the online test, the trained generator is used to achieve accurate channel estimation for the optimally designed pilots.

The pilot design lies in how to select the most informative position to insert the pilots in the time-frequency domain, which can be optimized as a feature selection problem. The Concrete Autoencoder (Concrete AE) was proposed in [15], which uses the concrete distribution and reparametrization trick to minimize the loss function to select features. However, traditional methods such as PCA or autoencoders use standard dimensionality reduction techniques to replace the original data features with new fewer features while ensuring maximum variance and minimum reconstruction loss. Such methods cannot directly select the original features [16]. Therefore, it cannot be directly used to reduce redundant features and reduce test costs. In the experimental simulation of the conference [15], the Concrete AE algorithm is compared with other feature selection algorithms such as PCA, MCFS [17] and the deep learning algorithm AEFS [18], and so on. Concrete AE shows better performance in finding the most informative features and removing the most redundancy. In this paper, we applied it to pilot design, Concrete AE can be used to select the most informative locations in the time-frequency grid to assign to pilots, thereby obtaining a near-optimal pilot pattern for each specific channel model. In network selection, we use concrete selector layers as the encoder, and the decoder part (also the generator part) consists of a DNN network.

cGAN is an extension of conventional GAN, both are an architecture based on the adversarial model, (i.e., discriminator) to train the generative model, (i.e., generator), the difference is that GAN’s generator learns the mapping from random noise to real data. However, this mapping is unstable and random, while cGAN is able to learn the mapping from conditional input to real data [19]. In the proposed scheme, the conditional input of the generator is the pilot optimized by the concrete selector, and the output is the estimated channel. Further taking the estimated channel and the true channel as the input of the discriminator, it can recognize the given input as a true label “1” or a false label “0”. To improve the channel estimation accuracy, the proposed scheme incorporates the MSE loss function in the loss function of cGAN.

## 3. System Model

### 3.1. Receive Signal

Consider a MIMO-OFDM system, there are *M_t_* transmit antennas and *M_r_* receive antennas. In the time-frequency resources of the NR air interface, it is assumed that one-time slot contains *N_s_* OFDM symbols, and one resource block group (RBG) contains *N_f_* subcarriers. Then the *i*th subcarrier signal of the *j*th OFDM symbol received by the *m_r_*th receiving antenna [20] can be expressed as

(1) $$y_{i,j,m_{r}}=\sum_{m_{t}=1}^{M_{t}}h_{i,j,m_{r},mt}w_{i,j,m_{r}}x_{i,j}+z_{i,j,m_{r}}$$

where $h_{{i,\;j,m}_{r}{,m}_{t}}$ and $w_{i,j,m_{r}}$ respectively represent channel coefficient and transmit precoder of the *m_t_*th transmit antenna, $x_{i,\;j}$ is the transmit signal, $z_{{i,\;j,m}_{r}}$ is additive white Gaussian noise (AWGN).

Since the pilot and the data use the same transmit precoder for beamforming, Equation (1) can be rewritten as

(2) $$y_{i,j,m_{r}}={\tilde{h}}_{i,j,m_{r}}x_{i,j}+z_{i,j,m_{r}}$$

where ${\tilde{h}}_{i,j,m_{r}}=\overset{M_{t}}{\underset{m_{t}=1}{\sum }}h_{{i,j,m}_{r}{,m}_{t}}w_{{i,j,m}_{r}}$ is defined as the precoding channel. Furthermore, for all OFDM symbols and subcarriers, the signal received by the *m_r_*th receiving antenna can be expressed as

(3) $$Y_{m_{r}}=H_{m_{r}}⊙X+Z_{m_{r}}$$

In the formula, $Y_{m_{r}}{,\;H}_{m_{r}},\;X$ and ${\;Z}_{m_{r}}$ are all *N_f_* × *N_s_* dimensional matrices, and the elements at the (*i, j*) position are respectively $y_{{i,j,m}_{r}}$, ${\tilde{h}}_{i,j,m_{r}}$, $x_{i,j}\;$ and $z_{{i,j,m}_{r}}$, ⊙  represents Hadamard product, which denotes the element-wise product.

### 3.2. Pilot Design Based on Concrete AE Network

Concrete AE network is an end-to-end differentiable method for global feature selection, which can effectively identify the feature subset with the largest amount of information, and use it for pilot design to select the most informative locations in the time-frequency grid, then the near-optimal pilot patterns for each specific channel model are available.

Assuming that the time-frequency grid used for selection is a noisy channel ***H****_noisy_*, the size of the grid is *N_f_ × N_s_.* In the offline training phase, ***H****_noisy_* can be obtained through the pilot frequency and the received signal, i.e., ***H****_noisy_* *= **Y**_mr_* ∅X, ∅ means dividing the corresponding elements of the two matrices. Then flattening the matrix ***H_noisy_*** to obtain its vectorized representation ***h****_noisy_* = [***h***_1_,***h***_2_, *…, **h**_D_*], where *D* = *N_f_* × *N_s_* is the vectorized time-frequency grid length. ***h****_noisy_* as the input of concrete AE network, correspondingly, the output of the encoder (concrete selector layer) is ***h****_p_,_noisy_*= [***h****_p_*_,1_***, h****_p_*_,2_,…, ***h****_p,L_*], *L* < *D*, ***h****_p,noisy_* is the most informative feature subset of ***h****_noisy_*, its *l*th element ***h****_p_*_,*l*_ is the output of the *l*th node of the specific selector layer, which can be expressed as

(4) $$h_{p,l}=h_{noisy}m_{l}$$

where *l* ∈ 1, …, *L*, $m_{l}={[m}_{l,1}{,m}_{l,2},\cdots {,m}_{l,D}{]}^{T}$ is a *D*-dimensional random variable sampled from a concrete distribution, the elements are defined as

(5) $$m_{l,j}=\frac{\exp\left(\log\alpha_{j}+g_{j}\right)/T}{\sum_{d=1}^{D}\exp\left(\left(\log\alpha_{d}+g_{d}\right)/T\right)}$$

In the above formula, $\alpha_{j}\in {\mathbb{R}}_{>0}^{D}$ is the concrete parameter, *T*∈(0,∞) is the temperature parameter, *g_j_* is sampled from a Gumbel distribution.

When *T* is close to 0, the concrete random variable will approach a discrete distribution, and the output vector $m_{l}\;$ will be approximated as a one-hot vector in probability $\alpha_{j}/\underset{p}{\sum }\alpha_{p}$ (only $m_{l,\;j}=1$, the remaining elements are zero). $\alpha_{j}$ and decoder weights ω can be iteratively updated by minimizing the following loss function

(6) $${ℒ}_{\mathrm{AE}}=\frac{1}{N}\sum_{n=1}^{N}∥f_{\omega }\left(h_{p,noisy}^{n}\right)-h_{noisy}^{n}{∥}_{2}^{2}$$

where, $∥\cdot {∥}_{2}$ represent 2-Norm, $f_{\omega }\left(\cdot \right)$ represents the decoder function, *N* is the number of training samples.

### 3.3. Channel Estimation Based on cGAN Network

After concrete AE network training, the selected pilots ${\;h}_{p,\;noisy}^{n}$ are input into the cGAN network. In the proposed scheme, the decoder of concrete AE and the generator of cGAN share the same network, the generator, and discriminator of cGAN both adopt the DNN network, and the generator of cGAN learns a mapping from ${\;h}_{p,\;noisy}^{n}$ to real channel ***h****_ideal_* (***h****_idea_*_l_ is the matrix $H_{m_{r}}$ after leveling 1 × *D* dimensional vectorized representation). During the offline training phase, the generator is responsible for estimating the vectorized channel $\overset{\wedge }{h}=[{\overset{\wedge }{h}}_{1},{\overset{\wedge }{h}}_{2},\cdots ,{\overset{\wedge }{h}}_{D}]$ from the conditional input $h_{p,\;noisy}^{n}$, and the discriminator can recognize the given input as a true label “1” or a false label “0”. After the training is successful, the trained generator can be used to perform channel estimation on the new pilot input.

The goal of cGAN is to let the generated channel fool the discriminator. At the same time, the discriminator needs to learn not to be cheated. A Nash equilibrium state is achieved by multiple alternating training of the two networks. To accomplish this optimization, the loss function of cGAN can be defined as

(7) $${ℒ}_{\mathrm{cGAN}}=\left(G_{\varphi },D_{\theta },h_{p,noisy},h_{ideal}\right)=E\left[\log D_{\theta }\left(h_{ideal}\right)+E\left[\log\left(1-D_{\theta }\left(G_{\varphi }\left(h_{p,noisy}\right)\right)\right)\right]\right]$$

where $D_{\theta }$ is the discriminator parameterized by *θ*, which aims to distinguish the generated channel from the real channel, *G_φ_* represents the generator parameterized by *φ*.

Hoping that the generated channel $\overset{\wedge }{h}$ is closer to the real channel $h_{ideal}$, so we need to minimize the *G_φ_* and maximize *D_θ_* in the loss function. Therefore, the cGAN objective function is

(8) $$\underset{G_{\varphi }}{\min}\underset{D_{\theta }}{\max}{ℒ}_{\mathrm{cGAN}}\left(G_{\varphi },D_{\theta },h_{p,noisy},h_{ideal}\right)$$

Furthermore, to ensure the correct direction of the generator optimization, the proposed scheme introduces an ${ℒ}_{2}$ loss function added to the cGAN loss as followed:

(9) $${ℒ}_{2}=Ε\left[||h_{ideal}-G_{\varphi }\left(h_{p,noisy}\right)|{|}_{2}^{2}\right]$$

Finally, the overall objective function can be defined as

(10) $$\underset{G_{\varphi }}{\min}\underset{D_{\theta }}{\max}\left({ℒ}_{\mathrm{cGAN}}\left(G_{\varphi },D_{\theta },h_{p,noisy},h_{ideal}\right)+{ℒ}_{2}\right)$$

## 4. Simulation Results and Analysis

In order to verify the channel estimation performance of the proposed CAGAN network under the MIMO-OFDM system, link-level simulation is employed in this experiment. Considering the air interface specification of the 5G system, in the time-frequency resources, a one-time slot contains 14 OFDM symbols, and there are 72 subcarriers in the frequency domain, which is equivalent to 6 RB (resource blocks), and each RB contains 12 subcarriers. The experimental channel environment adopts a Veh-A channel model with a frequency of 2.1 GHz, a bandwidth of 1.6 MHz, a delay spread of 1730 ns, and a user equipment (UE) speed of 50 km/h generated by a channel simulator developed by the University of Vienna [21]. In order to evaluate the performance of the proposed algorithm in different signal-to-noise ratio environments, the experiments based on the Veh-A channel model generate a number of different noise channel data between 0 and 30 dB for training and testing of the CAGAN network. Train and test datasets include 80,000 and 10,000 channel realizations respectively. We train all networks with a batch size of 256 utilizing the Adam optimizer, and this paper uses MSE (mean square error) as the evaluation index to measure the difference between the channel estimated and the real value.

For the part of the CAGAN network that acts as both the decoder in the autoencoder and the generator in the generative adversarial network, a six-layer fully connected deep neural network is used in the experiment, and the activation function of each hidden layer adopts LeakyReLU. The discriminator network of cGAN in the proposed scheme also uses a deep neural network, but the difference from the generative network is that the discriminator solves a binary classification problem. Therefore, the activation function of the output layer of the discriminator DNN network adopts the sigmoid function.

**Figure 2:**
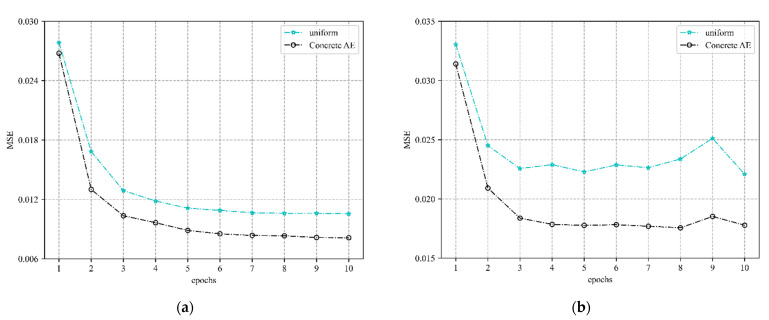
Performance comparison between uniform pilot design and concrete AE pilot optimization; (**a**) number of pilots = 16; (**b**) number of pilots = 8.

The first step of the CAGAN scheme is to use concrete AE to realize the pilot optimization design. Figure 2 shows the channel estimation performance comparison between the concrete AE network for pilot optimization and the traditional equal-spaced pilot design. The simulation results show that the estimation accuracy of the concrete AE optimized pilot method is higher than that of the traditional uniformly spaced pilot method, and the smaller the number of pilots, the more obvious the improvement of the channel estimation accuracy by pilot optimization as shown in Figure 2b. This also shows that the channel estimation method combined with a concrete AE network for pilot optimization design has significant advantages in saving pilot overhead and improving system spectral efficiency for massive MIMO systems.

**Figure 3:**
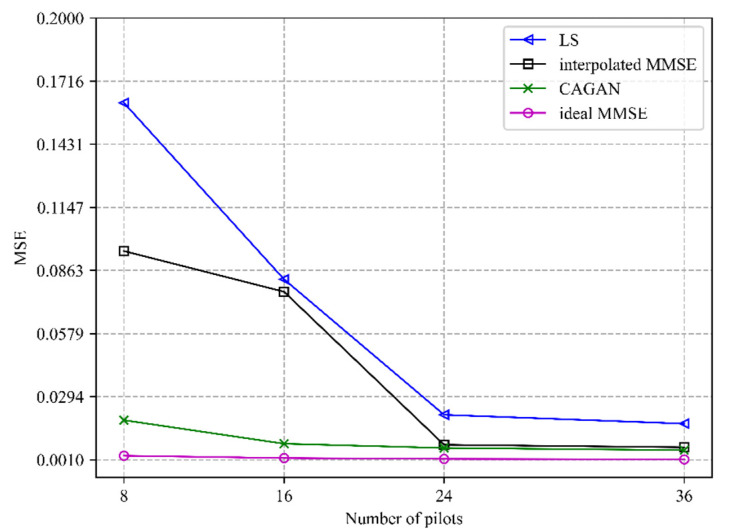
Comparison of channel estimation performance of different algorithms under different pilot numbers when SNR = 15 dB.

In addition to improving the accuracy of channel estimation, the application of pilot design has another important role in reducing pilot overhead. In this experiment, we simulate the channel estimation accuracy comparison between CAGAN and traditional algorithms with different numbers of pilots under the same signal-to-noise ratio. The specific performance is shown in Figure 3.

It can be seen that the accuracy of each algorithm gradually decreases with the increase in the number of pilots. Because the ideal MMSE algorithm only estimates the channel information of the pilot positions where is inserted, the estimation accuracy of the algorithm hardly changes with the number of pilots. The CAGAN algorithm proposed in this paper achieves a higher estimation accuracy when the number of pilots is eight than the traditional algorithm LS when the number of pilots is forty-eight; secondly, compared with the MMSE algorithm, the CAGAN only needs fewer pilots to achieve the same estimation accuracy. Therefore, the proposed CAGAN algorithm can effectively save pilot overhead in channel estimation.

**Figure 4:**
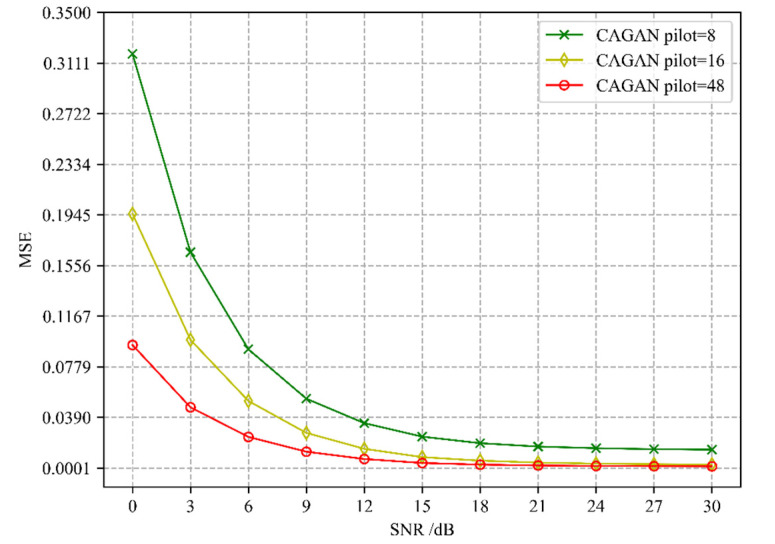
Comparison of CAGAN channel estimation accuracy with different pilot numbers.

The second step of the CAGAN scheme is to use cGAN for channel estimation based on the optimized pilots of concrete AE. In addition to the influence on the accuracy due to the location of the pilots, (i.e., whether to adopt an optimized pilot scheme), the number of different pilots is another important factor. Figure 4 presents the comparison of the channel estimation accuracy of CAGAN when the number of pilots is 8, 16, and 48, respectively. As we can find that with the increase in the number of pilots, the estimation accuracy increases accordingly. When SNR > 18 dB, for a time-frequency grid resource containing 1008 RE (resource elements), the estimation accuracy is basically the same when the number of pilots is 16 and 48, and even when the number of pilots is 8, the accuracy can also reach 10^−3^ to 10^−4^ magnitude. It can be seen that the CAGAN scheme has better robustness to the number of pilots at a light higher signal-to-noise ratio, and can effectively improve the spectral efficiency of the system under the premise of ensuring the accuracy of channel estimation.

**Figure 5:**
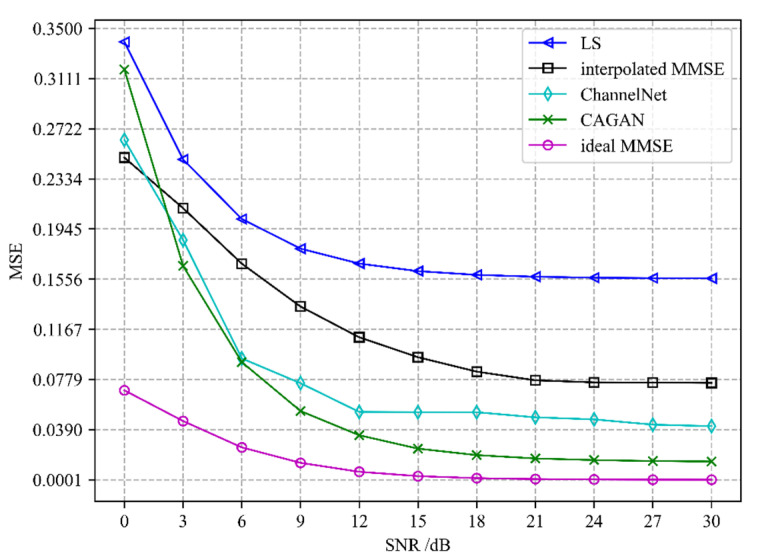
Performance comparison of different algorithms when the number of pilots is 8.

**Figure 6:**
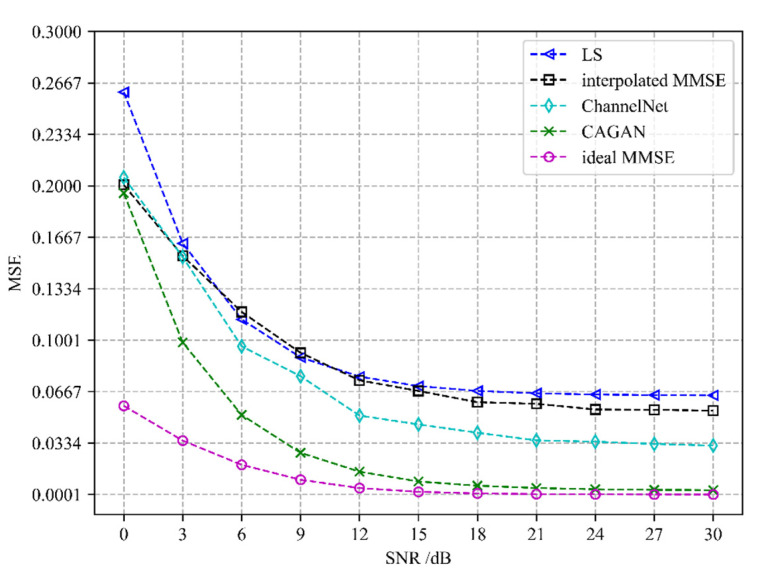
Performance comparison of different algorithms when the number of pilots is 16.

Different channel estimation algorithms’ performance evaluation is depicted in Figure 5 and Figure 6. We compare the CAGAN with four algorithms: least squares (LS), interpolated minimum mean square error (interpolated MMSE), ideal minimum mean square error (ideal MMSE), and ChannelNet proposed in [22]. All experiments are under exactly the same experimental setting. Ideal MMSE only estimates the channel information of the pilot position at the receiving end and does not estimate the complete time-frequency grid channel information. An ideal MMSE requires a second-order statistical channel and noise variance as prior information, which is impractical in actual communication, so it is taken as the upper bound of performance evaluation.

It can be seen from Figure 5 and Figure 6 that the performance of the CAGAN algorithm proposed in this paper is significantly better than the traditional LS and MMSE algorithms, reflecting the advantages of deep learning algorithms; when the SNR > 15 dB, the estimation performance of each algorithm tends to be stable, and the estimation accuracy of ChannelNet is between 10^−1^ and 10^−2^, its performance is still worse than CAGAN. When the number of pilots is 16, under the same SNR condition, CAGAN has obvious advantages over the other three algorithms, the estimation accuracy is improved to between 10^−3^ and 10^−4^, and under high signal-to-noise ratio, CAGAN and ideal MMSE is much closer.

**Figure 7:**
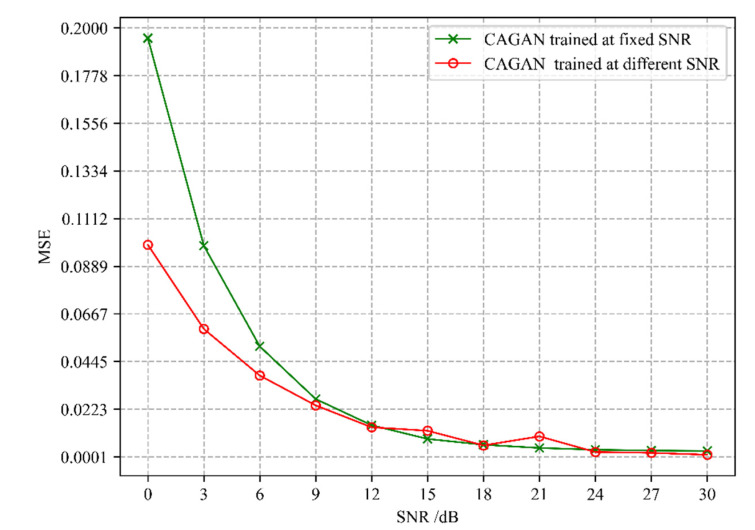
Performance comparison of CAGAN in two training modes.

In the training phase of the CAGAN network, we tried two different training modes. The first is CAGAN trained at fixed SNR, that is, the noisy channel data with SNR = 15 dB is used to input the CAGAN network. After the training is completed, in the testing phase, the successfully trained models are used to predict the noisy channel data with the SNR value range of 0~30 dB and the step size of 3 dB. The second mode is CAGAN trained at different SNR, the noisy channel data with the SNR value range of 0~30 dB and the step size of 3 dB is trained and tested in turn. The simulation results of the two training modes are shown in Figure 7. It can be seen from the figure that when the SNR > 9 dB, the channel estimation performance of the two modes tends to be consistent, but the first method can greatly reduce the training time and complexity of the algorithm because it only needs to be trained once. Additionally, experiments also show that the algorithm has better robustness against noise.

In this experiment, we use the application programming interface (API) of Keras to calculate and analyze the complexity of different neural networks. Trainable parameters and FLOPs represent the number of parameters and computations required by the network, the specific values are shown in Table 1. It can be seen that the complexity of the CAGAN network proposed in this paper is higher than that of ChannelNet, but this is because ChannelNet does not use a deep neural network for pilot selection and post-interpolation. When the neural network for interpolation is added, the complexity of the ChannelNet2 network in the table is much higher than that of CAGAN. In addition, we noticed that applying a convolutional neural network instead of a fully connected neural network will greatly reduce the complexity of the network, but this may lose some performance. Therefore, considering that the hybrid network architecture proposed in this paper realizes two functions of pilot optimization and channel estimation, and the final experimental performance has been significantly improved, the increase in its complexity is also acceptable.

## 5. Conclusions

Aiming at the problem of FDD downlink channel estimation in MIMO-OFDM systems, we utilize the newly exploited CACAN network structure to study the channel estimation in this paper. The core idea of the whole algorithm is to use the excellent feature extraction ability of concrete AE to find the most suitable pilot position, and the accurate estimation of the channel is further completed by the generative adversarial network. The simulation results prove that the CAGAN scheme has lower pilot overhead, higher estimation accuracy, and stronger robustness against noise, and can be applied to MIMO-OFDM systems in more complex application scenarios.

## Figures and Tables

**Table 1:** The complexity analysis of different DL Networks.

Methods	Number of Flops	Number of Parameters
CAGAN	2557 K	1281 K
CAGAN (CNN replace DNN)	548 K	275 K
ChannelNet	1731 K	865 K
ChannelNet2	3903 K	1954 K

## Data Availability

Not applicable.
